# On “Light”

**Published:** 1896-06

**Authors:** Myles Standish

**Affiliations:** Boston, Mass.


### ON "LIGHT." [1]

BY MYLES STANDISH, M.D., BOSTON, MASS.

MR. PRESIDENT AND GENTLEMEN,—When I received the notice stating that I was to read a paper, I felt that I was sailing under false colors, as I had made no preparations to read a paper, and had no time in which to prepare one. I hardly knew what to do, but decided to come and say a few practical words which may be of interest to you.

---

[1] Read before the American Academy of Dental Science, January 1, 1896.

I do not intend to talk on "light" from a scientific point of view, nor do I propose to enter into the mathematics or chemistry of the subject,—I am simply prepared to talk on it as it most directly and practically interests us, and purely from a clinical point of view, as the subject presents itself to the oculist rather than to the scientist.

By light we mean the light that we all use in our work day and night, and I will begin with artificial light. Let me bring to your mind two or three small facts in regard to the physiology of the eye. You will recall the statement which you heard when at school, that "the eye is a photographic camera." As you were then told, the retina serves as a sensitive plate and literally makes a picture every time we look at anything, and then with inconceivable rapidity the picture is dissolved when we look somewhere else. This is made possible through the peculiar functional action of a fluid called the "retinal purple." Not very much is known about the process, except that, in some mysterious manner, there is deposited in the retina a photographic picture in this purple, which purple soon becomes exhausted and new purple is created for the next picture. There is a limit, however, to the creation of this purple, a fact very easily shown in the condition known as "snow-blindness." If you have ever read of men who have been walking over fields or prairies, especially in the colder climates, where they are sometimes compelled to walk on the snow for weeks or months at a time, you have learned that they have been afflicted with a red-blindness,—that is, everything after a while began to look red, and at night when the sun went down, or when it became cloudy, they were unable to see at all. This redness is due to this: that so much light has come in through the pupil that the retinal purple is completely exhausted, and if you go on long enough under such circumstances you destroy the eye. Now, here is an important fact with regard to the chemistry of light: that this retinal purple, under ordinary circumstances, is being replaced as fast as exhausted, unless, of course, it is taxed entirely beyond its powers. We have certain habits and methods of using our eyes, and it is easy to see that the lower parts of the eye are more used to receiving light than the parts that are above. The brows protect the eye from the light coming from above, but we have no such protection from the light coming from below, and we, therefore, experience great inconvenience from the reflection of the sun shining upon the water or upon a field of snow. I had a chance to demonstrate this to my satisfaction at one time when I went up to the White Mountains on a vacation in the middle of winter. I took up with me a variety of dark glasses for the purpose of trying their effect, and walking across the fields one morning, which were absolutely white with a few inches of newly-fallen snow, I tried these various glasses. I found the smoked glasses the most comfortable, the blue next, and the green the least. I then remembered a conversation I had had with Professor Goodale, the botanist, at Cambridge, in which he told me of his experience in some country, New Zealand, I think, where they went over a glacial formation or a white stony formation, or something of that sort, which was very white, and the Indian guides told them to blacken their cheeks and lower eyelids, and the light would not hurt them. They did this, and they found that the reflection of the light from the white surface did not affect them. Now, I could not see why blackening the cheeks had anything to do with the eye, but I went back to the hotel and blackened my under eyelids, and found that I could walk across the snow-field and keep my eyes open just as well as if I had on a pair of smoked glasses. Thinking that perhaps I had gotten used to the snow, I went back again and wiped off the burnt cork and found that the snow was trying just as it was in the first place, and I then came to the conclusion that the reason the light hurt me was because it came from the wrong direction. I relate this to you simply to show that the effect of light on the visual purple is greater under unaccustomed conditions.

Until quite recently, I do not know but always, the oculist has said that artificial light required three qualities,—steadiness, proper color, and intensity. And I think that most oculists, and almost everybody else, felt that the last quality was the most important of the three. When I found I was announced to come here and talk on this subject, I thought I would see what the authorities had to say on the subject of the effect of light upon the eyes, and I looked through the text-books on physiology, the ophthalmological text-books, and a fairly extensive library of books of reference, including the "Encyclopædia Britannica," to see if I could find something that I could use, but I could find nothing bearing directly on this subject, and, therefore, I was obliged to get my matter out of my own head, the result of my clinical experience in taking care of the eyes of people who complain.

Within a short time we have had introduced the arc light, the incandescent electric light, and the incandescent gas light. The arc light came first, and it was hailed with great delight for the lighting of our streets, and has since been used in the large retail dry-goods and other stores; but they soon found that in the stores it played great mischief with the eyes of the employés. We then said it was due to its brilliancy. Well, I think it was, to a certain extent, but to a greater extent this was due to its variability; it would go up and down and sputter, and it was this unsteadiness that caused the poor clerks to suffer so intensely with their eyes. And they are not the only ones who are injured from this inconvenience. I think it is a most outrageous thing that we should permit in our streets these electric arc lights to be hung almost directly in front of the eyes of the people as they walk along. When they are put up on a high pole, twenty to thirty feet above the ground, it is not so bad, but when you come to place them in front of a shop-window, not more than three or four feet above your head as you walk along, I say it is an injury to everybody that walks upon the street at night. If you walk up Washington Street, say as far as Dover, just as they are turning on the lights for the night, they usually do this before it gets very dark, you will find that although these lights are plentifully sprinkled along in front of the show-windows, when first turned on you do not notice them very much. If, however, you take the same walk about eight o'clock in the evening, you will find that the lights which you did not notice earlier will be boring into your eyes; they will trouble you, and if you have sensitive eyes, they will harm you. Now, the intensity of that light was just as great at four or five o'clock as it was after dark, and, of course, the intensity is greater than that of daylight, and yet we did not notice the lights at all until we came upon them and perhaps heard them sputter, and why not? My explanation is that our eyes, in the first instance, were adapted to receiving much light and the intensity of the electric light was not noticed, but after dark when our eyes were adjusted to the darkness and the brilliant light suddenly came before us, the effect on the visual purple and other percipient apparatus was very injurious.

But, passing by the arc lights, which do not concern us very much in our present discussion, because we do not have to use them in our work, we come to the incandescent lights. When the incandescent electric light was first introduced, it was regarded by oculists everywhere as a great, good thing. You had a light which met all the requirements,—it was steady, it was clear, and it had great brilliancy, and, therefore, it must be a fine light to work by. And I remember that for many years I recommended it as being the best light, and when people came in and told me that it was not, I thought they must certainly be wrong, because the incandescent light was steady and had great power, and I was convinced that the people did not know what they were talking about. But it occasionally happens that our patients do know something about the cause of their complaints and sometimes more than we know ourselves. After I had had numerous complaints from book-keepers and others employed in offices and manufacturing places where they used the electric light, I came to the conclusion that perhaps the light really did hurt their eyes, and I started to investigate. The first case that brought this matter seriously to my attention was that of a girl, whom I saw at the infirmary. She said that she had been employed at the Mechanics Building during the fair, and had worked at a table on which an incandescent light was placed, and there she worked for two or three months with this light always in the same position, and she said that she was sure the trouble with her eyes was the result of the light. I did not say much, though it was plainly evident that something had badly affected the deeper-seated coatings of the eye, to the extent that she had practically lost the sight of it. My next case was that of a young man, who was employed in a broker's office to write down the stock quotations. You know they have a big black-board with the names of the various stocks on it, and the duties of this young man were to look at the " ticker" and mark the numbers showing the rise and fall of stocks on the black-board. This board was in a dark corner of the office and an incandescent light hung directly before him, so that all day long he was standing in a definite position and the light fell on a definite spot of his eye, and the result was choroiditis, and he lost the sight of his eye.

The cases began to multiply in which patients complained of the incandescent electric light, and I began to think perhaps it was not so good a light as it might be, and I started out to find out what I could about it. The first place I visited was the composing-room of one of our great metropolitan newspapers in which they had incandescent lights. In some way I had become the fashion in that composing-room, having had fifteen or sixteen patients in succession, and on inquiring about the lights that were used they said it was the incandescent electric, and I said that I thought that ought to be the best light they could have, but they replied that I ought to come down about the middle of the evening when everything was turned on and see what I thought of them. I went down and instantly saw that the incandescent electric light did not always have at least one of the qualities that I thought it had,—that is, steadiness. It seems that this newspaper company had put in an electric plant of their own, and in order to lighten the cost to themselves had let to their neighbors lights or power, or both, and they had run their dynamo for all it was worth. Their machine was overloaded, with the result that the lights were constantly going up and down, and I decided that it was not the light but the flickering condition that made all the mischief there. By and by I had another case of inflammation in the eye, caused by the incandescent electric light. This was a man who had a desk in a dark office, and he had an electric light hanging down alongside of him, and by this light he worked all day with the result that he practically lost the sight of his eye, and then I began to think about it, and it occurred to me that our old friend, the visual purple, might have something to do with the difficulty.

Now, I remember a year or two ago of being in a hotel one night and the electric light was in such a position that I was obliged to look up at it a few seconds as I was in the act of turning it off. I retired, but right opposite my bed there was a door, the upper half of which was one large pane of ground glass which was illuminated by the lights in the entry. To my surprise, I saw on that ground glass a dozen or fifteen dark horseshoes. Now that showed me immediately that I had received sufficient illumination in looking up at that light to almost exhaust the retina for visual purposes over the areas covered by these dark horseshoes. Being of an inquisitive turn of mind, I took up my watch, which I always keep in a convenient place when I go to bed, and I found that the deepest one of those images did not disappear until nearly twelve minutes had gone by. Of course that was a test under peculiarly advantageous circumstances, but it shows that a light which is so intense as to exhaust the retinal purple in such a manner that it was not replaced for about twelve minutes, must be a dangerous light, and a room that is lighted by incandescent lights in chandeliers hanging down low, with the plain glass globes and the bare fibre burning into your eyes, is badly lighted.

I have hung up here some lights that I want to show you and say a word about them. They are two electric lights of the same power, about fifty candle-power, I think, and I want to hold them up for you to look at and see how you like them. When I put this plain one up, you get an image of the fibre,—the illumination that comes from that light comes entirely from that minute fibre,—in other words, you get as much illumination as you get from several gas lights all from that slender film; therefore, the intensity of the illumination must increase as to the smallness of its source, and that is where the mischief comes. Such a light as that has no business to be put anywhere so that its image will fall on the eye. Now, this other light has the same power, but you will notice it has a frosted bulb. It gives just as much light as the other, less about 3 per cent.,—that is, you get 97 per cent. of the light that you get from this plain one, but you get it from the full size of the bulb, and the same amount of illumination originating from a bulb as big as that has a very different intensity on the spot that it covers on the retina than it does when it comes from this small fibre. If some one will please put out the lights, we will try the effect of these two lights in a darkened room. This light with the plain glass bulb when I held it up a moment ago, gave you some annoyance to look at, but look at it now. You find that it annoys you a great deal more now in this dark room, and there are hardly any of you who care to look at it even for a few seconds. This one with the frosted bulb you can look at with comparative ease, and the reason of it is simply that here the whole bulb is the source of the illumination, while in the case of the plain glass, just that little film is the source of illumination.

We will now consider another light, the incandescent gas light, the so called Wellsbach light. That has the same qualities as the ordinary electric light. It has great intensity and greater whiteness, if anything, and steadiness. It has more steadiness than the incandescent electric light has in our ordinary country service, and more than it has on our Back Bay service, or at least as that service was until they put on their big storage plant. I asked them at the Back Bay Electric Works why they could not keep their lights at the same degree of intensity, and they said the trouble was that the current was so intermittently used,—that is, a hotel would take a considerable amount of it, and then, perhaps, it might not be used again for a quarter of a mile, and sometimes there were extra amounts used without notification, so that to keep their current steady seemed to be an impossibility unless it was more generally and evenly used. They hope to get better effects now that they have a storage battery, which they intend to use as a sort of a balance-wheel to supply the deficiency of their motors and dynamos when they are called suddenly to deliver a great deal of electricity.

To return to this Wellsbach light, it is an incandescent light of astonishing whiteness and brilliancy. It has most of the qualities of the other lights, and is steadier. If you will put out the lights once more, you will see illustrated the very thing that we had shown with the two electric lights which I held up to you, only on a larger scale. Here is a very bright light, and it is a light that none of us want to watch a great deal; so that, even with that great brilliancy, it is not an ideal light for general illumination, but for certain purposes it is a wonderfully good light, and those purposes are near work, reading, etc., and even in those cases we must use it in a proper manner. With this shade put on we have a light that the average layman and a good many oculists think is a beautiful light to read by. It is so brilliant that you can almost see the fibre of the paper, and the average layman sits down and reads by it, feeling that he has the best possible light he can have. Now, how does that compare with the conditions that were presented to you a few moments ago? We now have a room that is very dark, and in that room we have a space that is brilliantly illuminated, and, with our eyes adjusted to this general darkness, we take up a sheet of white, calendered paper, and sit down and calmly look at it, perhaps for several hours, and when these facts are stated broadly one must conclude that it is a very bad thing to do. The eye is adapted here for darkness and yet we have a spot here, right in the middle of the eye, the very spot that you are using most, that is constantly fixed on a very bright light reflected by the paper from the Wellsbach light. Now, supposing you do want to read with that light, if you will please turn on the electrics again, you will see a highly proper state of affairs, and a very comfortable one for the use of the eyes,—the dazzling brilliancy of the single light is gone. If you have enough illumination so that the room to the eyes is light, then we have a light which is peculiarly well adapted to the purposes for which we want it. To have the thing ideal when you place a pencil on the face of your book you should have two shadows, one coming from the general illuminating light, and the other from the light by your side, and the shadow from your reading light should be a trifle darker than the one which comes from your general light. One thing more, the light of course should be to your left and at your back in reading, writing, or anything of that kind. The reason is this: If I arrange to have my light come from the right, all that I write is in shadow, and I am watching the formation of the letters in a shadow which constantly falls on the very point I wish to see most distinctly. The same principle applies for any other sort of work, you must have the shadows fall so that you can see what you are doing, and not have the shadows fall upon it. I also have here a scheme which the Wellsbach people wanted me to bring here to-night, a dark cap which is put on underneath the light to modify it. Well, if your light is as powerful as this one is, I think it would be necessary to use it when it is new, because you could not get sufficiently brilliant illumination in your room to make the proportions what they should be, but these lights deteriorate after a little while, so that later on you could use it with this ordinary white cap, or none at all for that matter.

Now, to come back to my text, we will leave the artificial light and come back to daylight, which is the light I presume you gentlemen work by mostly. Everybody has said, because the artists say so (and I think very rightly for their purposes), that the north light is the best light to work by, but the artists' work is such that they do not want the sun. Now, a light without sunlight lacks certain qualities. The north light softens the outlines of things, and consequently I am not at all sure that a north light is the best light for many mechanical purposes. If you get sunlight, the shadows falling from it have very much sharper edges. I do not know enough about dentistry to form an accurate idea of the light you require, but it seems to me that a south light or a west light, if properly screened, would be just as good, or even better, than a north light for your purposes. Now, there comes in right here a very important point, and that is the question how we judge distances. This is something that very few of us have ever given any thought to and it seems to be somewhat of an instinctive action. The child has to learn to judge distance, for when it first begins to observe, the things at the other end of the table seem to be within his reach until he learns by experience. We judge depths by shadows. If you have a cavity, and the light is from one side, the depth of that cavity is judged by the angle with which the light falls across the bottom of it. That is done instinctively, and it seems to me—at least from a theoretical stand-point—that you would get a sharper shadow from an extreme south or west light.

But there is another important fact that I would call your attention to, and that is the utter disastrousness of cross lights in judging depths. If you put a patient so that the cavity into which you are looking is illuminated from two sources of light of equal strength, you would get a shadow from each side, and where they cross you would have your deepest shadow, so that that particular point would always appear to be the deepest point of the cavity. If you cannot judge depth without being obliged to look at it very closely then you are simply fatiguing your eyes. The man who attempts to do mechanical work in a cross light comes to grief.

It requires much extra exertion to judge depth in a cross light, the eyes being focussed up to their utmost standard every minute, and the effect may be to ultimately cause near-sightedness. The value of the shadow in all mechanical work is something that is not thoroughly understood. One of the great electric light companies in this city got an idea that they would furnish the rooms in which their people were doing fine mechanical work with the best possible light, so they fitted up their draughting and engineers' offices, etc., by painting the ceiling white, and then they put in an arc light; then they put a shade underneath that, the upper side of which was also painted white, and the result of this whole scheme was that the light was thrown up on the ceiling and diffused down, and they had a brilliant, white light, but to their utter astonishment every one complained of the light, and inside of two months two of the four draughtsmen engaged there were patients of mine.

If you had gone down to see the light they were working in you would have found yourself in a very brilliantly lighted room. The whole air would have seemed radiant with light, but you would not have found a shadow in that room,—you couldn't make a shadow,—and the result was that the draughtsmen, who had been accustomed to judging when they had reached a certain point by the angle made between the shadow and the pen or pencil, found themselves obliged to look closely for every point to which they drew a line, and their eyes were kept under a heavy strain all the time, and naturally they soon gave out. Therefore, shadows are not without great value in judging distances.

I do not know that I have anything more to say; I think I have exhibited all my crankiness on the subject, but if I have impressed it upon you gentlemen to look out for your eyes, to get a proper illumination both for your work and in your houses, and if also I could make enough impression on the community at large that electric arc lights should not be hung in the streets a few feet above our heads as we walk along,—and not a few of them with bright red globes, which is a combination that I will guarantee will destroy any man's eyes who looks at it much,—I shall have accomplished something which will be a great boon to the community in the long run.

